# A healthy lifestyle attenuates the effect of polypharmacy on total and cardiovascular mortality: a national prospective cohort study

**DOI:** 10.1038/s41598-018-30840-9

**Published:** 2018-08-22

**Authors:** David Martinez-Gomez, Pilar Guallar-Castillon, Sara Higueras-Fresnillo, Jose R. Banegas, Kabir P. Sadarangani, Fernando Rodriguez-Artalejo

**Affiliations:** 1 Department of Physical Education, Sport and Human Movement, Universidad Autónoma de Madrid, Madrid, Spain; 2 Department of Preventive Medicine and Public Health, Universidad Autónoma de Madrid/ IdiPaz, CIBER of Epidemiology and Public Health (CIBERESP), Madrid, Spain; 3 IMDEA Food Institute. CEI UAM+CSIC, Madrid, Spain; 4 School of Physiotherapy, Faculty of Health Sciences, Universidad San Sebastián, Lota 2465, Santiago, 7510157 Chile; 5 Escuela de Kinesiología, Facultad de Salud y Odontología, Universidad Diego Portales, Santiago, 8370109 Chile

## Abstract

This work examines whether the increased all-cause and cardiovascular disease (CVD) mortality associated with polypharmacy could be offset by a healthy lifestyle. We included a prospective cohort of 3,925 individuals representative of the Spanish population aged ≥60 years, who were recruited in 2000–2001 and followed up through 2014. Polypharmacy was defined as treatment with ≥5 medications. The following lifestyle behaviors were considered healthy: not smoking, eating a healthy diet, being physically active, moderate alcohol consumption, low sitting time, and adequate sleep duration. Individuals were classified into three lifestyle categories s: unfavorable (0–2), intermediate (3–4) favorable (5–6). Over a median 13.8-y follow-up, 1,822 all-cause and 675 CVD deaths occurred. Among individuals with polypharmacy, intermediate and favorable lifestyles were associated with an all-cause mortality reduction (95% confidence interval [CI]) of 47% (34–58%) and 54% (37–66%), respectively; 37% (9–56%) and 60% (33–76%) for CVD death, respectively. The theoretical adjusted hazard ratio (95%CI) associated with replacing 1 medication with 1 healthy lifestyle behavior was 0.73 (0.66–0.81) for all-cause death and 0.69 (0.59–0.82) for CVD death. The theoretical adjusted hazard ratio (95%CI) for all-cause and CVD mortality associated with simply reducing 1 medication was 0.88 (0.83–0.94) and 0.83 (0.76–0.91), respectively. Hence, adherence to a healthy lifestyle behavior can reduce mortality risk associated with polypharmacy in older adults.

## Introduction

Improvements in the effectiveness and coverage of health care, including pharmacological treatments, have substantially contributed to recent gains in health expectancy in old age in high-income countries^1^. However, prevention and management of age-related chronic conditions have resulted in an increased number of medications taken by older adults^2,3^. Polypharmacy, defined as the use of multiple medications or the administration of an excessive number of medications^4^, is very frequent in the elderly and has been associated with many adverse health outcomes, including poor treatment adherence, adverse drug reactions, drug-drug interactions, drug-disease interactions, falls, fractures, frailty, hospitalizations, physical and cognitive functional impairments, and medical errors which, in turn, increase the risk of death^5^. In addition, costs of drug treatments have risen sharply in recent decades, and represent a challenge for healthcare systems sustainability^6,7^. Unfortunately, there is little high-quality evidence to date on how to reduce polypharmacy, with deprescribing being the main strategy^8–10^.

A healthy lifestyle has been shown to improve health in older adults^11,12^. Several healthy lifestyle behaviors, including not smoking, a healthy diet, being physically active, moderate alcohol consumption, less sedentariness and adequate sleep duration have well-documented positive effects on mortality in the elderly; in fact, it seems that it is never too late to obtain some benefits from adopting healthy lifestyle behaviors^13–18^. Therefore, it is of interest to assess whether the adverse mortality effect of polypharmacy could be attenuated by a healthy lifestyle in the old age. These findings might represent an important shift in the approach to provide health in among older adults because the objectives go beyond preventing deaths exclusively through pharmacology treatments. Hence, we used data from a cohort of older adults in Spain to examine whether the increased all-cause and cardiovascular disease (CVD) mortality associated with polypharmacy could be offset by a healthy lifestyle. Additionally, we assessed the theoretical mortality benefits that could be obtained by replacing medications with healthy lifestyle behaviors in individuals with polypharmacy.

## Methods

### Study design and participants

We analyzed data from a cohort of 4,008 individuals, representative of the non-institutionalized population aged ≥60 years in Spain^16,19^. The study participants were recruited in 2000/2001 using probabilistic and multistage cluster sampling. The clusters were initially stratified according to region of residence and size of municipality. Next, census sections and households were successively chosen randomly within each cluster. Study participants were finally selected in sex and age strata. The final study participation rate was 71%. Data were collected by home-based personal interview and physical examination, performed by trained personnel. All methods were carried out in accordance with relevant guidelines/regulations. The study was approved by the Clinical Research Ethics Committee of the *La Paz* University Hospital in Madrid, Spain. All participants provided written informed consent.

### Exposure variables

During the home visit, participants were asked to show all prescribed and over-the-counter medications that they were currently taking. We recorded up to ten medications, and the total number of drugs was classified into three categories: 0–1 medications, 2–4 medications and ≥5 medications or polypharmacy in the current study^20^.

We considered six lifestyle behaviors that were self-reported with standardized and validated questionnaires^21–26^: (i) smoking, (ii) diet, (iii) alcohol consumption, (iv) physical activity, (v) sedentary behavior and (vi) sleep duration. According to published evidence and lifestyle recommendations^12–18^, the following lifestyle behaviors were considered healthy: not smoking (never smoking or having quit >15 years ago), eating a healthy diet (index ≥median in the cohort), being physically active (very/moderately active), moderate alcohol intake (≤20 and ≤ 30 g/d in female and male drinkers, respectively), low sedentariness (≤7 h/d), and adequate sleep duration (7–8 h/d). A healthy lifestyle score was defined as the number of healthy lifestyle behaviors, and individuals were classified into three categories: unfavorable (0–2 healthy lifestyle behaviors), intermediate (3–4 healthy lifestyle behaviors), and favorable (5–6 healthy lifestyle behaviors).

### Ascertainment of mortality

The outcome variables were all-cause and CVD mortality from the study baseline through the end of follow-up at December 31, 2014. The number, cause and date of deaths were obtained by a computerized search of the National Death Index of the Ministry of Health and the vital registry of the National Institute for Statistics. The vital status was successfully ascertained for 99.9% of the cohort. The underlying cause of death was determined by a nosologist according to the International Classification of Diseases, Tenth Edition, with cardiovascular death corresponding to codes I00-I99.

### Other variables

Sex, age, and educational level were recorded. Body weight, height and waist circumference were measured and body mass index (BMI) was calculated as kg/m^2^ ^19^. Blood pressure readings were performed in the right arm at the level of the heart using standardized methods^27^. Hypercholesterolemia was defined as an affirmative answer to the following question: Has your doctor ever told you that you have high (blood) cholesterol? Cognitive function was assessed with the Mini-Mental State Examination^28,29^. Agility and mobility limitations were defined as an affirmative answer to standardized questions^30^. Finally, we registered the following diseases diagnosed by a physician and reported by the study participant: chronic lung disease, CVD, diabetes mellitus, cancer at any site, and depression.

### Statistical analyses

Of the 4,008 study participants, 83 were excluded because of missing data on lifestyle behaviors or other variables, so the final sample comprised 3,925 individuals (2,214 women). Pearson correlation was used to analyze the relationship between the number of medications and healthy lifestyle behaviors.

Cumulative all-cause and CVD mortality according to medication and healthy lifestyle behaviors were calculated using the Kaplan-Meier method, and differences were assessed with log-rank tests. We also used Cox proportional hazards regression, with days as the time scale, to estimate the hazard ratios (HRs) and 95% confidence intervals (CIs) for all-cause and CVD mortality associated with medication or healthy lifestyle behaviors. The analyses took 0–1 medications or a favorable lifestyle, respectively, as the reference category. The dose-response relationship was tested with a *P* for trend, calculated by modeling the number of medications and healthy lifestyle behaviors as continuous variables; we also used restricted cubic spline regression to graphically illustrate this relationship.

Next, to examine whether healthy lifestyle behaviors attenuate the effect of medications on all-cause or CVD mortality we initially estimated the association between healthy lifestyle behaviors and mortality outcomes stratified by medication categories. Also, we checked the potential modifier effect of medications on the associations between healthy lifestyle behaviors and mortality outcomes by including an interaction term in Cox models. Then, we assessed the association of the combined exposure to healthy lifestyle behaviors and medications with mortality by modelling nine categories of exposure (that is, 3 medication categories ×3 healthy lifestyle behaviors categories) and participants taking 0–1 medications with a favorable lifestyle was the reference category –hypothetically the healthiest category.

Finally, the theoretical all-cause and CVD mortality benefit from reduced polypharmacy, achieved by replacing 1 medication with 1 healthy lifestyle behavior, was estimated in a way similar to isotemporal/isocaloric substitution models^31,32^ among participants taking ≥5 medications. Since these methods of analysis assume a constant amount of “discretionary time or calories”, we considered that the total number of medications and healthy lifestyle behaviors represented a constant amount of “discretionary options”. The theoretical mortality benefits of deprescribing 1 medication to reduce polypharmacy was operationalized as decreasing any medication and tested by reversing the order in the medication exposure variable (i.e., decreasing the number of medications taken) among older adults taking ≥5 medications. We also calculated a risk advancement period, which shows the difference in survival in persons with a varying number of medications or healthy lifestyle behaviors that is equivalent to the all-cause or CVD mortality associated with each 1-year increase in chronological age^16^.

Analyses were first adjusted for sex and age (model 1), and subsequently for educational attainment, BMI, waist circumference, systolic blood pressure, hypercholesterolemia, Mini-Mental State Examination score, agility limitation, mobility limitation, chronic lung disease, CVD, cancer, diabetes mellitus, and depression (model 2). These covariates were selected according to previous works in the same research area^5^. Multicollinearity detection was examined in all analyses, but variance inflation factors were in the normal range.

We assessed the assumption of proportionality of hazards both graphically and by testing the significance of the interaction of medication or healthy lifestyle with time of follow-up, and we found no evidence of departure from such assumption (all P for interaction > 0.1). Statistical significance was set at two-sided P < 0.05. Analyses were performed with STATA® v.14.1 for Macintosh.

## Results

Descriptive characteristics of the study participants at baseline by medication and healthy lifestyle categories are reported in Table 1. Overall, compared to participants with 0–1 medications or with a favorable lifestyle, those taking ≥5 medications or with an unfavorable lifestyle were older and more frequently women, and had lower education, more functional and cognitive limitations, and more chronic diseases. There was only a weak correlation, albeit significant, between the number of medications and healthy lifestyle behaviors (*r* = −0.15, *P* < 0.001).

**Table 1:** Characteristics of the participants at baseline, by categories of medication and healthy lifestyle.

	Medication category			*P*	Healthy lifestyle category			*P*
	0–1	2–4	≥5		Unfavorable	Intermediate	Favorable	
*n*	1295	1787	843		665	2308	952	
Women, %	48.8	58.6	63.6	<0.001	57.9	57.4	53.0	0.028
Age, years	70.7 ± 8.0	72.0 ± 7.8	73.6 ± 7.9	<0.001	75.4 ± 8.5	71.7 ± 7.8	69.9 ± 7.0	<0.001
Educational attainment, %								
No education	48.0	51.1	59.8		64.6	52.6	41.6	
Primary	36.1	36.8	29.9		26.1	34.7	42.2	
Secondary or higher	15.9	12.1	10.3	<0.001	9.4	12.7	16.2	<0.001
Body mass index, kg/m^2^	28.3 ± 4.1	29.0 ± 4.5	29.6 ± 4.8	<0.001	28.9 ± 5.3	28.9 ± 4.4	28.7 ± 4.0	0.265
Waist circumference, cm	97.3 ± 11.2	98.8 ± 12.1	100.8 ± 12.5	<0.001	99.8 ± 13.9	98.5 ± 11.6	98.6 ± 11.5	0.067
Systolic blood pressure, mm Hg	142.3 ± 18.5	144.1 ± 18.8	142.6 ± 21.0	0.465	144.7 ± 21.0	143.3 ± 19.0	141.8 ± 18.4	0.003
Hypercholesterolemia, %	17.8	25.6	32.6	<0.001	19.4	25.0	27.1	0.001
MMSE, score	25.8 ± 4.3	25.5 ± 4.5	24.9 ± 4.5	<0.001	23.6 ± 5.4	25.5 ± 4.4	26.6 ± 3.3	<0.001
Agility limitations, %	42.5	60.5	79.8	<0.001	76.9	59.3	44.5	<0.001
Mobility limitations, %	34.7	52.2	73.9	<0.001	75.7	50.9	34.2	<0.001
Chronic lung disease, %	7.0	14.5	25.4	<0.001	19.5	15.0	9.1	<0.001
Cardiovascular disease*, %	2.1	7.6	21.1	<0.001	13.7	8.3	6.1	<0.001
Diabetes, %	4.9	15.7	30.4	<0.001	19.1	14.5	14.7	0.065
Cancer, %	1.3	1.8	2.5	0.058	2.2	1.9	1.3	0.744
Depression, %	3.4	10.7	20.1	<0.001	15.3	10.4	6.7	<0.001

Values are means ± SD or %. MMSE: Mini-Mental State examination.

Over a median follow-up of 13.8 years (5^th^ to 95^th^ percentile: 1.9–14.2) a total of 1,822 deaths occurred, 675 of them due to cardiovascular disease. In Kaplan-Meier analyses, cumulative all-cause and CVD deaths progressively increased with the number of medications and decreased from unfavorable through favorable categories of lifestyle (all log-rank tests *P* < 0.001) (Figure S1).

After multivariable adjustment (model 2, Table 2), and compared to taking 0–1 medications, the adjusted HR (95%CI) of all-cause death was 1.32 (1.15–1.51) for 3–4 medications and 1.75 (1.49–2.06) for ≥5 medications; corresponding results for CVD mortality were 1.50 (1.17–1.92) and 2.11 (1.60–2.78). In contrast, those with intermediate and favorable *vs* unfavorable lifestyle had an adjusted HR (95%CI) of 0.58 (0.51–0.67) and 0.48 (0.40–0.57) for all-cause mortality, and of 0.55 (0.43–0.70) and 0.45 (0.32–0.59) for CVD mortality (Table 2). Both medications and healthy lifestyles showed a clear dose-response with mortality (Figure S2); the adjusted HR (95%CI) for all-cause and CVD mortality associated with 1-medication increase were 1.12 (1.09–1.14) and 1.15 (1.10–1.19), respectively, and with 1-healthy lifestyle behavior increase were 0.81 (0.77–0.85) and 0.78 (0.72–0.85), respectively (all *P* for trend < 0.001) (Table 2). These associations remained virtually the same after adjustment for the other exposure variable (Table S1).

**Table 2:** All-cause and cardiovascular disease (CVD) mortality risk according to number of medications and healthy lifestyle behaviors in older adults.

	*n*	All-cause mortality HR (95%CI)			CVD mortality HR (95%CI)		
		Cases	Model 1	Model 2	Cases	Model 1	Model 2
Medication category							
0–1 medications	1295	452	1 (Reference)	1 (Reference)	137	1 (Reference)	1 (Reference)
2–4 medications	1787	842	1.38 (1.20–1.57)	1.32 (1.15–1.51)	316	1.64 (1.29–2.08)	1.50 (1.17–1.92)
≥5 medications	843	528	2.03 (1.75–2.34)	1.75 (1.49–2.06)	222	2.68 (2.09–3.43)	2.11 (1.60–2.78)
*P* for trend			<0.001	<0.001		<0.001	<0.001
Per 1-medication increase			1.14 (1.11–1.16)	1.12 (1.09–1.14)		1.19 (1.15–1.23)	1.15 (1.10–1.19)
Healthy lifestyle category							
Unfavorable	665	469	1 (Reference)	1 (Reference)	195	1 (Reference)	1 (Reference)
Intermediate	2308	1027	0.55 (0.48–0.63)	0.58 (0.51–0.67)	374	0.50 (0.40–0.63)	0.55 (0.43–0.70)
Favorable	952	326	0.43 (0.37–0.51)	0.48 (0.40–0.57)	106	0.36 (0.27–0.48)	0.45 (0.32–0.59)
*P* for trend			<0.001	<0.001		<0.001	<0.001
Per 1-healthy lifestyle increase			0.79 (0.75–0.82)	0.81 (0.77–0.85)		0.74 (0.68–0.80)	0.78 (0.72–0.85)

Model 1 adjusted for sex and age.

Model 2 adjusted as in model 1 and for educational attainment, body mass index, waist circumference, systolic blood pressure, hypercholesterolemia, Mini-Mental State Examination, agility limitations, mobility limitations, chronic lung disease, CVD, cancer, diabetes mellitus, and depression.

**Figure 1:**
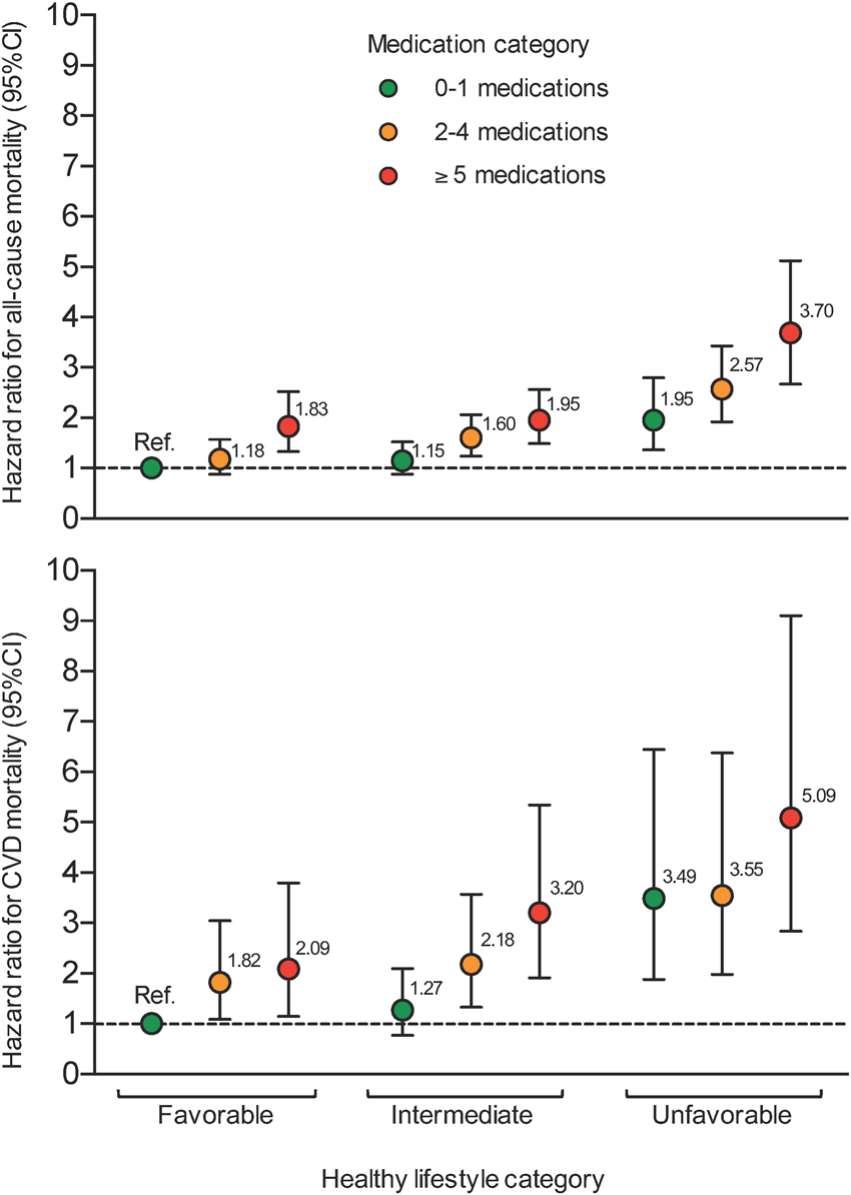
All-cause and cardiovascular disease (CVD) mortality risk across categories of medication and healthy lifestyle in older adults (*n* = 3925). Analyses were adjusted as model 2 in Table 2. Number of participants in each increasing category from the X-axis segment was as follows: 354 (Ref.), 452, 146, 794, 1031, 483, 147, 304, and 214.

The association between heathy lifestyle behaviors and mortality did not vary by medication category (all P for interactions >0.1). Among participants with polypharmacy (≥5 medications), an intermediate or favorable lifestyle was associated with an all-cause mortality reduction (95%CI) of 47% (34–58%) and 54% (37–66%), respectively; for CVD death, figures were 37% (9–56%) and 60% (33–76%) (Table 3). In combined analyses, adherence to a healthier lifestyle attenuated the increased all-cause and CVD mortality associated with taking 2–4 medications and polypharmacy remarkably (Fig. 1 and Table S2). Also, as compared to participants with 0–1 medications and a favorable lifestyle, those with ≥5 medications and an unfavorable lifestyle had the highest risk of all-cause death (HR = 3.70, 95%CI: 2.67–5.12) and CVD death (HR = 5.09, 95%CI: 2.84–9.10) (Fig. 1).

**Table 3:** All-cause and cardiovascular disease (CVD) mortality risk according to healthy lifestyle in older adults stratified by medication category in older adults.

	*n*	All-cause mortality HR (95%CI)			CVD mortality HR (95%CI)		
		Cases	Model 1	Model 2	Cases	Model 1	Model 2
With 0–1 medications							
Unfavorable lifestyle	147	93	1 (Reference)	1 (Reference)	43	1 (Reference)	1 (Reference)
Intermediate lifestyle	794	267	0.63 (0.45–0.89)	0.63 (0.45–0.88)	72	0.41 (0.25–0.67)	0.42 (0.26–0.70)
Favorable lifestyle	354	92	0.55 (0.37–0.80)	0.56 (0.38–0.83)	22	0.34 (0.18–0.63)	0.39 (0.20–0.74)
*P* for trend			0.002	0.005		<0.001	0.001
Per 1-healthy lifestyle increase			0.85 (0.76–0.94)	0.85 (0.76–0.95)		0.71 (0.59–0.86)	0.73 (0.60–0.89)
With 2–4 medications							
Unfavorable lifestyle	304	207	1 (Reference)	1 (Reference)	81	1 (Reference)	1 (Reference)
Intermediate lifestyle	1031	478	0.59 (0.49–0.71)	0.62 (0.51–0.75)	175	0.57 (0.39–0.82)	0.57 (0.40–0.83)
Favorable lifestyle	452	157	0.43 (0.34–0.54)	0.45 (0.35–0.58)	60	0.46 (0.29–0.74)	0.47 (0.30–0.75)
*P* for trend			<0.001	<0.001		<0.001	<0.001
Per 1-healthy lifestyle increase			0.78 (0.73–0.83)	0.79 (0.74–0.85)		0.77 (0.68–0.86)	0.78 (0.69–0.88)
With ≥5 medications							
Unfavorable lifestyle	214	159	1 (Reference)	1 (Reference)	71	1 (Reference)	1 (Reference)
Intermediate lifestyle	483	282	0.52 (0.41–0.64)	0.53 (0.42–0.66)	127	0.57 (0.41–0.80)	0.63 (0.44–0.91)
Favorable lifestyle	146	77	0.46 (0.35–0.61)	0.46 (0.34–0.63)	24	0.36 (0.22–0.57)	0.40 (0.24–0.67)
*P* for trend			<0.001	<0.001		<0.001	0.002
Per 1-healthy lifestyle increase			0.80 (0.74–0.87)	0.80 (0.74–0.87)		0.78 (0.69–0.88)	0.81 (0.70–0.92)

Model 1 adjusted for sex and age.

Model 2 adjusted as in model 1 and for educational attainment, body mass index, waist circumference, systolic blood pressure, hypercholesterolemia, Mini-Mental State Examination, agility limitations, mobility limitations, chronic lung disease, CVD, cancer, diabetes mellitus, and depression.

Among those with polypharmacy, the adjusted mortality HR (95%CI) associated with replacing 1 medication with 1 healthy lifestyle behavior was 0.73 (0.66–0.81) for all-cause death and 0.69 (0.59–0.82) for CVD death (Figure S3); it is approximately equivalent to the reduced mortality associated with being 3 years younger. This suggests that replacement might have greater benefits than deprescribing, because the adjusted HR (95%CI) for all-cause and CVD mortality associated with simply reducing 1 medication was 0.88 (0.83–0.94) and 0.83 (0.76–0.91), respectively (both *P* for trend < 0.001); this is a mortality equivalent to a 1–1.5 years younger age.

## Discussion

In a population-based cohort of older adults in Spain, adherence to a healthier lifestyle was linked to a reduction of all-cause and CVD mortality associated with polypharmacy. Specifically, among individuals with polypharmacy, those with a favorable lifestyle showed a 54% and 60% lower all-cause and CVD death risk, respectively, than those with an unfavorable lifestyle. In addition, the theoretical effect of replacing medications with healthy lifestyle behaviors evinced a greater reduction of mortality than simply deprescribing the number of medications. Since taking medications and adherence to a healthy lifestyle are not related in the old age, these findings are especially important for older adults, which usually take an excessive number of medications, and therefore, public health and clinical strategies are needed to improve their health behaviors.

Although many clinical trials have shown that drug treatments improve survival in different clinical scenarios, we found that the number of medications taken had a direct dose-response relationship with mortality from all causes and CVD –the leading cause of death among older adults. Individuals with ≥5 medications had about two times higher risk of death (HR = 1.75, 95%CI: 1.49–2.06 for all-cause mortality and HR = 2.11, 95%CI: 1.60–2.78 for CVD mortality) than those with none or only 1 medication. Our results are in line with most previous research on this topic, regardless of the cut-point selected to define polypharmacy^5^. In fact, studies with better adjustment for the confounding effect of morbidity found a clearer association between the number of medications taken and mortality^5^. Polypharmacy, therefore, can be considered a “red flag” for high mortality risk in older adults. Consequently, the increased risks of overtreatment by too much medication is a public health message that should be disseminated throughout society, given the widespread tendency to self-administer over-the-counter medications^33–36^.

In our study, adherence to a healthier lifestyle partially offsets the increased mortality associated with polypharmacy. Interestingly, even an intermediate lifestyle (i.e., 3–4 out of 6 healthy lifestyle behaviors) showed a significant reduction in the increased mortality; this, and the observed dose-response reduction of mortality with a higher number of healthy lifestyle behaviors, indicates that each of them “counts” for a certain benefit. In fact, older adults with polypharmacy who adhered to a favorable lifestyle had an excess mortality which was similar or even lower to that of those with 0–1 medications and an unfavorable lifestyle for all-cause and CVD mortality, respectively (Fig. 1).

On the other hand, participants taking 2–4 medications also had an increased all-cause (HR = 1.32, 95%CI: 1.15–1.51) and CVD (HR = 1.50, 95%CI: 1.17–1.92) mortality risk in our cohort. These older adults, especially those who take 4 medications, are at risk for polypharmacy, and to avoid its initiation, adherence to healthier lifestyles would be essential and is supported by our results. For example, among individuals taking 2–4 medications, those with a favorable lifestyle had a 55% and 53% lower all-cause and CVD death risk, respectively, than did those with an unfavorable lifestyle (Table 3); they even had a lower mortality risk than those taking none or 1 medication and adherence to an unfavorable lifestyle (Fig. 1).

Deprescribing is the process of tapering, stopping, discontinuing, or withdrawing medications, supervised by a healthcare professional (e.g., general physicians, nurses, pharmacists), with the goal of reducing polypharmacy^8,9^. Although it is feasible and safe^8^, two recent meta-analyses on the effect of deprescribing found no convincing evidence of benefits in mortality^8,9^. Page *et al*.^8^ reported that, in nonrandomized studies, deprescribing to reduce polypharmacy significantly decreased all-cause mortality (OR = 0.32, 95%CI: 0.17–0.60), but this was not statistically significant in randomized trials (OR = 0.82, 95%CI 0.61–1.11). Moreover, Johansson *et al*.^9^ found that deprescribing polypharmacy had no effect on all-cause mortality (OR = 1.02, 95%CI: 0.84–1.23). Both reviews also indicated that the quality of the evidence was low and that more effective strategies to reduce polypharmacy should be developed and assessed^8,9^. In our study, we examined the impact of replacing medications with healthy lifestyle behaviors; thus, this included the joint effect of “deprescribing drug treatments” and “prescribing a healthy lifestyle” as compared with only deprescribing. While we acknowledge that the effect of medical advice on adopting healthy lifestyle behaviors in healthcare settings could be modest^37,38^, our results suggest that the benefit of replacement could be 2 to 3 times higher than that of only deprescribing a drug treatment; for example, while replacing 1 medication with 1 healthy lifestyle behavior was linked to a 27% reduction of all-cause mortality, reducing 1 medication was associated with only a 12% reduction. Thus a healthy lifestyle is “medicine” and may replace other medications. However, it must be noted that these findings are theoretical because when “deprescribing” some considerations such as harmful effects, the stage of the disease, doses, the importance of this medication at short- medium- and long-term, and individual barriers, could not be taken into account in our study.

This study has several strengths, including a large representative sample of community-dwelling older adults that allows for certain generalization of results. Also, study variables were collected by trained staff using standardized methods. Moreover, analyses were adjusted for a substantial number of confounders, including chronic morbidity. Despite these strengths, our findings should be interpreted in the context of the following methodological limitations. First, since this is an observational study, there might be some residual confounding, and associations may not imply causation. However, the strength and dose-response of the associations found, the consistency across medication categories, and a long follow-up -which makes reverse causality less likely- all lend credence to our study results and support causal inference. Second, information on drug treatments and lifestyle behaviors was obtained at baseline. Our analyses assumed that medication exposure and lifestyle behaviors have certain stability over time, but some changes are still possible and would likely shift the observed associations toward the null. Third, lifestyle behaviors were self-reported and dichotomized, which may have led to recall bias and non-differential misclassification; again, this could have underestimated the impact of polypharmacy and lifestyle on mortality. Fourth, although the analyses were adjusted for several confounding variables, some potential confounders were not included due to they were not available in our study (e.g., dietary patterns, family health history, blood samples). In addition, we calculated a propensity score in *post hoc* analyses with our 12 covariates, which have been suggested as a potential confounder in pharmacoepidemiology^39^, and the associations between polypharmacy and mortality did no change.

In conclusion, adherence to a healthy lifestyle may help to reduce the increased risk of all-cause and CVD death associated with polypharmacy in older adults. In addition, replacing medications with healthy lifestyle behaviors may be theoretically a useful strategy to reduce polypharmacy and maximize health benefits, as compared to only deprescribing. However, this strategy should be directly tested in clinical trials which, in addition to mortality, focus on other outcomes relevant for older people, such as frailty, falls, and cognitive and physical decline.

## Electronic supplementary material

Supplementary file
