# Supplementary material for: A healthy lifestyle attenuates the effect of polypharmacy on total and cardiovascular mortality: a national prospective cohort study
Source: Sci Rep. 2018 Aug 22;8:12615. doi: 10.1038/s41598-018-30840-9 (PMC6105613; doi:10.1038/s41598-018-30840-9)
Supplement: Supplementary file 1 — Supplementary file [file 41598_2018_30840_MOESM1_ESM.docx]

**A healthy lifestyle attenuates the effect of polypharmacy on total and cardiovascular mortality: a national prospective cohort study**

Supplementary file

Table S1. Independent all-cause and cardiovascular disease (CVD) mortality risk according to number of medications and healthy lifestyle behaviors in older adults

|  |  |  | All-cause mortality  HR (95%CI) |  |  | CVD mortality  HR (95%CI) |
| --- | --- | --- | --- | --- | --- | --- |
|  | *n* | Cases |  |  | Cases | Model 1 |
| **Medication category** |  |  |  |  |  |  |
| 0-1 medications | 1295 | 452 | 1 (Reference) |  | 137 | 1 (Reference) |
| 2-4 medications | 1787 | 842 | 1.33 (1.15-1.53) |  | 316 | 1.51 (1.19-1.93) |
| ≥5 medications | 843 | 528 | 1.77 (1.50-2.08) |  | 222 | 2.13 (1.62-2.80) |
| *P* for trend |  |  | <0.001 |  |  | <0.001 |
| Per 1-medication increase |  |  | 1.12 (1.09-1.14) |  |  | 1.14 (0.72-0.85) |
| **Healthy lifestyle category** |  |  |  |  |  |  |
| Unfavorable | 665 | 469 | 1 (Reference) |  | 195 | 1 (Reference) |
| Intermediate | 2308 | 1027 | 0.59 (0.52-0.68) |  | 374 | 0.57 (0.45-0.72) |
| Favorable | 952 | 326 | 0.48 (0.40-0.58) |  | 106 | 0.44 (0.32-0.60) |
| *P* for trend |  |  | <0.001 |  |  | <0.001 |
| Per 1-healthy lifestyle increase |  |  | 0.82 (0.77-0.85) |  |  | 0.78 (1.09-1.19) |

Analyses were adjusted for age, sex, educational attainment, body mass index, waist circumference, systolic blood pressure, hypercholesterolemia, Mini-Mental State Examination, agility limitations, mobility limitations, chronic lung disease, CVD, cancer, diabetes mellitus, depression, and medications for the healthy lifestyle analysis or healthy lifestyle behaviors for the medication analysis.

Table S2. All-cause and cardiovascular disease (CVD) mortality risk across c number of medications and healthy lifestyle behaviors categories in older adults

| **Healthy lifestyle category** | **Medication category** | All-cause mortality  HR (95%CI) |  | CVD mortality  HR (95%CI) |
| --- | --- | --- | --- | --- |
| Favorable | 0-1 medications | 0.27 (0.20-0.37) |  | 0.20 (0.11-0.35) |
| Favorable | 2-4 medications | 0.31 (0.24-0.41) |  | 0.25 (0.16-0.38) |
| Favorable | ≥5 medications | 0.53 (0.37-0.75) |  | 0.69 (0.41-1.15) |
|  |  |  |  |  |
| Intermediate | 0-1 medications | 0.32 (0.24-0.42) |  | 0.36 (0.23-0.55) |
| Intermediate | 2-4 medications | 0.43 (0.34-0.55) |  | 0.43 (0.30-0.62) |
| Intermediate | ≥5 medications | 0.69 (0.54-0.89) |  | 0.70 (0.46-1.05) |
|  |  |  |  |  |
| Unfavorable | 0-1 medications | 0.50 (0.42-0.67) |  | 0.41 (0.25-0.67) |
| Unfavorable | 2-4 medications | 0.53 (0.42-0.67) |  | 0.63 (0.44-0.89) |
| Unfavorable | ≥5 medications | 1 (Reference) |  | 1 (Reference) |

Analyses were adjusted for age, sex, educational attainment, body mass index, waist circumference, systolic blood pressure, hypercholesterolemia, Mini-Mental State Examination, agility limitations, mobility limitations, chronic lung disease, CVD, cancer, diabetes mellitus, and depression.

Figure S1. Kaplan-Meier curves for cumulative all-cause and cardiovascular disease (CVD) mortality in older adults, by categories of medication and healthy lifestyle

Figure S2. All-cause and cardiovascular disease (CVD) mortality risk according to number of medications and healthy lifestyle behaviors in older adults. Analyses were obtained from restricted cubic spline regressions and adjusted for age, sex educational attainment, body mass index, waist circumference, systolic blood pressure, hypercholesterolemia, Mini-Mental State Examination, agility limitations, mobility limitations, chronic lung disease, CVD, cancer, diabetes mellitus, and depression. Solid lines indicate hazard ratios and dashed lines indicate 95% confidence intervals. Y-axis segments shown in log scale.


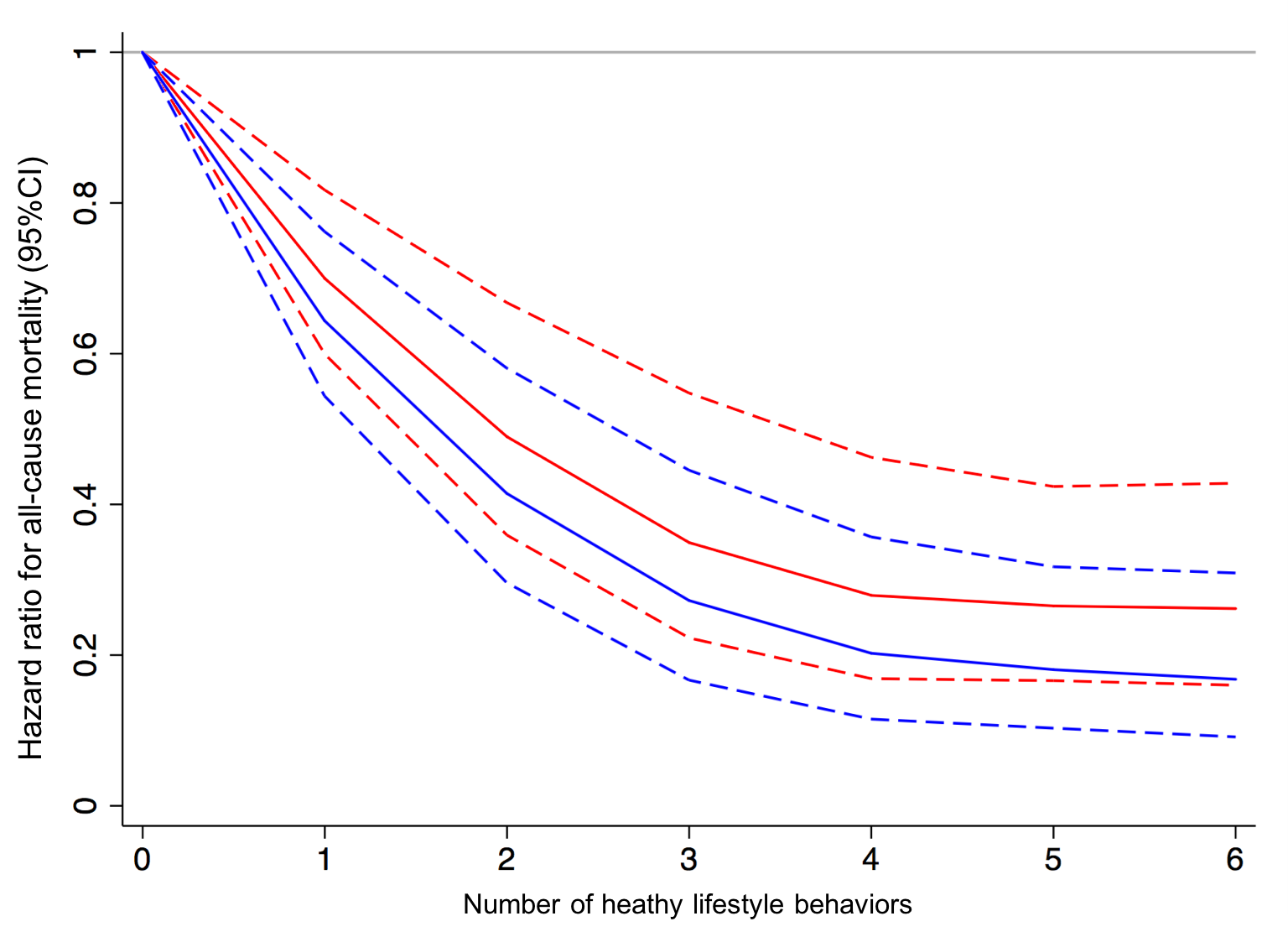

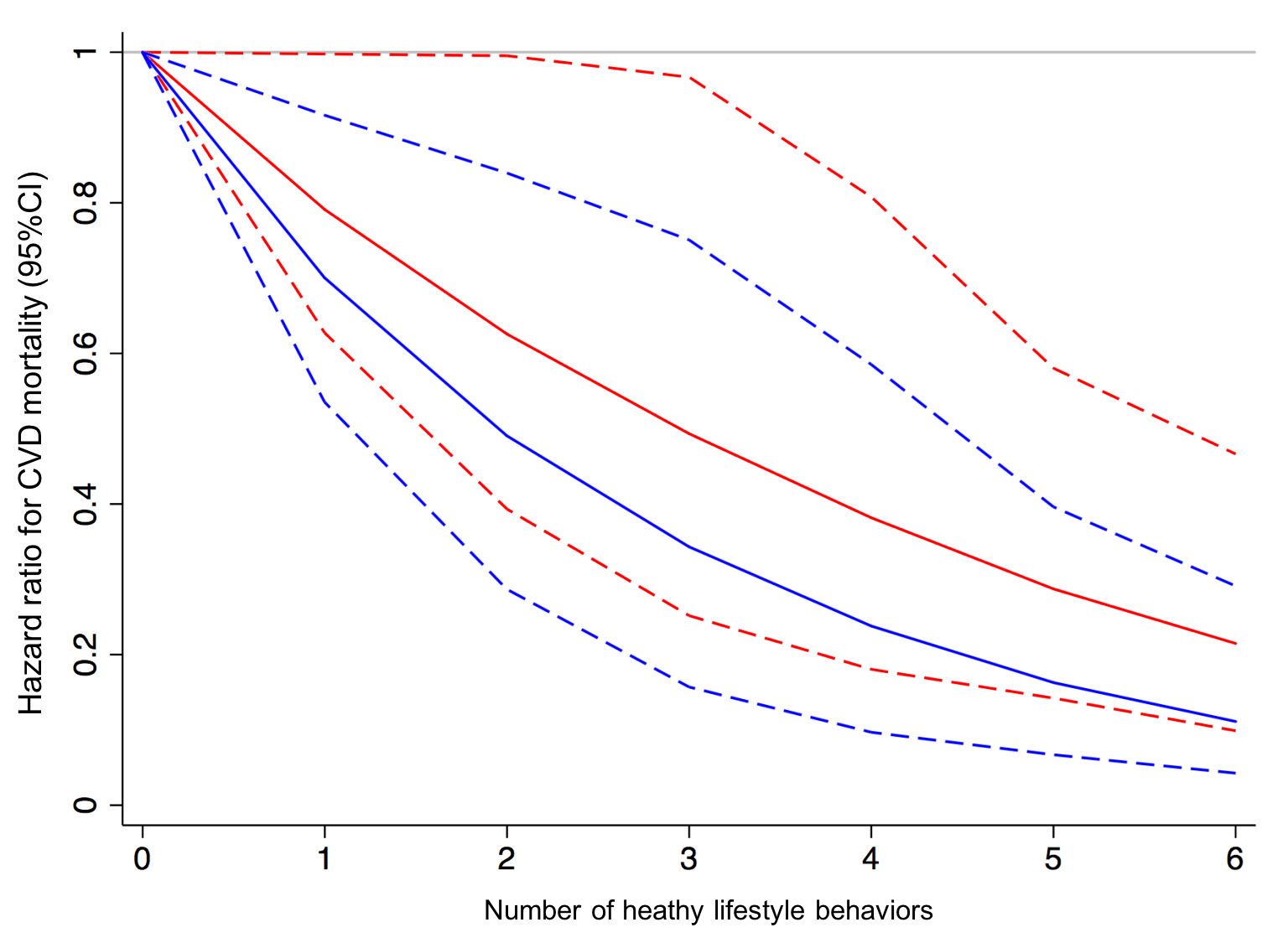


Individual (red) and substitution (blue) all-cause and cardiovascular disease (CVD) mortality risk according to the number of healthy lifestyle behaviors in older adults with polypharmacy. Analyses were obtained from restricted cubic spline regressions and adjusted for age, sex educational attainment, body mass index, waist circumference, systolic blood pressure, hypercholesterolemia, Mini-Mental State Examination, agility limitations, mobility limitations, chronic lung disease, CVD, cancer, diabetes mellitus, and depression. Solid lines indicate hazard ratios and dashed lines indicate 95% confidence intervals
